# Polymyxin Resistance Among XDR ST1 Carbapenem-Resistant *Acinetobacter baumannii* Clone Expanding in a Teaching Hospital

**DOI:** 10.3389/fmicb.2021.622704

**Published:** 2021-03-26

**Authors:** Letícia Dias de Melo Carrasco, Andrei Nicoli Gebieluca Dabul, Camila Maria dos Santos Boralli, Gabriela Marinho Righetto, Iago Silva e Carvalho, Janaína Valerini Dornelas, Camila Pacheco Silveira Martins da Mata, César Augusto de Araújo, Edna Mariléa Meireles Leite, Nilton Lincopan, Ilana Lopes Baratella da Cunha Camargo

**Affiliations:** ^1^Laboratory of Molecular Epidemiology and Microbiology, Department of Physics and Interdisciplinary Science, São Carlos Institute of Physics, University of São Paulo, São Paulo, Brazil; ^2^Hospital Risoleta Tolentino Neves, Belo Horizonte, Brazil; ^3^Department of Microbiology, Institute of Biomedical Sciences, University of São Paulo, São Paulo, Brazil

**Keywords:** carbapenem-resistant *Acinetobacter baumannii*, polymyxin resistance, extensively drug-resistant, pan drug-resistant, *A. baumannii* ST1, *A. baumannii* ST79, PmrC, PmrB

## Abstract

*Acinetobacter baumannii* is an opportunistic pathogen primarily associated with multidrug-resistant nosocomial infections, for which polymyxins are the last-resort antibiotics. This study investigated carbapenem-resistant *A. baumannii* strains exhibiting an extensively drug-resistant (XDR) phenotype, including four isolates considered locally pan drug-resistant (_L_PDR), isolated from inpatients during an outbreak at a teaching hospital in Brazil. ApaI DNA macrorestriction followed by PFGE clustered the strains in three pulsotypes, named A to C, among carbapenem-resistant *A. baumannii* strains. Pulsotypes A and B clustered six polymyxin-resistant *A. baumannii* strains. MLST analysis of representative strains of pulsotypes A, B, and C showed that they belong, respectively, to sequence types ST1 (clonal complex, CC1), ST79 (CC79), and ST903. Genomic analysis of international clones ST1 and ST79 representative strains predicted a wide resistome for β-lactams, aminoglycosides, fluoroquinolones, and trimethoprim-sulfamethoxazole, with *bla*_*OXA–23*_ and *bla*_*OXA–72*_ genes encoding carbapenem resistance. Amino acid substitutions in PmrB (Thr232Ile or Pro170Leu) and PmrC (Arg125His) were responsible for polymyxin resistance. Although colistin MICs were all high (MIC ≥ 128 mg/L), polymyxin B MICs varied; strains with Pro170Leu substitution in PmrB had MICs > 128 mg/L, while those with Thr232Ile had lower MICs (16–64 mg/L), irrespective of the clone. Although the first identified polymyxin-resistant *A. baumannii* strain belonged to ST79, the ST1 strains were endemic and caused the outbreak most likely due to polymyxin B use. The genome comparison of two ST1 strains from the same patient, but one susceptible and the other resistant to polymyxin, revealed mutations in 28 ORFs in addition to *pmrBC*. The ORF codifying an acyl-CoA dehydrogenase has gained attention due to its fatty acid breakdown and membrane fluidity involvement. However, the role of these mutations in the polymyxin resistance mechanism remains unknown. To prevent the dissemination of XDR bacteria, the hospital infection control committee implemented the patient bathing practice with a 2% chlorhexidine solution, a higher concentration than all *A. baumannii* chlorhexidine MICs. In conclusion, we showed the emergence of polymyxin resistance due to mutations in the chromosome of the carbapenem-resistant *A. baumannii* ST1, a high-risk global clone spreading in this hospital.

## Introduction

*Acinetobacter baumannii* is an opportunistic pathogen often associated with hospital-acquired infections worldwide, especially in intensive care units (ICUs) (Gundi et al., 2009; Zhang et al., 2013; Lin and Lan, 2014). *A. baumannii* has high rates of resistance to multiple antimicrobials due to its propensity to rapidly acquire resistance genes or even due to intrinsic resistance mechanisms typical to the genus (Peleg et al., 2008; Durante-Mangoni and Zarrilli, 2011). Furthermore, *A. baumannii* growth structured as biofilms on medical devices and mucous surfaces difficult their elimination, contributing to cause persistent and recurrent infections (Rodríguez-Baño et al., 2008). Epidemiologically, *A. baumannii* is one of the most severe ESKAPE pathogens (*Enterococcus faecium, Staphylococcus aureus, Klebsiella pneumoniae, A. baumannii, Pseudomonas aeruginosa*, and *Enterobacter* spp.) (Boucher et al., 2009). In addition, carbapenem-resistant *A. baumannii* is on the top of the World Health Organization (WHO) list of critical priorities for developing new antimicrobials (Tacconelli et al., 2018).

Polymyxins (colistin and polymyxin B) are antibiotics from the 1960s reintroduced in the 2000s in an attempt to treat infections caused by multidrug-resistant (MDR) gram-negative bacteria, particularly those resistant to carbapenems, as last-resort antibiotics (Karaiskos and Giamarellou, 2014). Combination therapy, such as polymyxins/aminoglycosides or meropenem or tigecycline, is considered when *A. baumannii* is resistant to carbapenem but susceptible to these antibiotics (Satlin et al., 2020). However, the use of tigecycline is not recommended or at least controversial (Ni et al., 2016). On the other hand, the meropenem/colistin combination improves survival in critically ill patients infected with carbapenem-resistant *A. baumannii* (Park et al., 2019). Since their reintroduction into clinical practice, polymyxins still display good activity against non-fermentative pathogens (Dias et al., 2016); however, resistance has increased in recent years (Karaiskos and Giamarellou, 2014).

At first, the polymyxin resistance mechanisms were only chromosomally related and, therefore, more difficult to disseminate (Cai et al., 2012). Later, plasmid-mediated genes conferring resistance to polymyxins were described, the so-called *mcr* genes. Initially, researchers thought that the *mcr* genes were only among *Enterobacterales* (Fernandes et al., 2016; Liu et al., 2016, 2017; Xavier et al., 2016; Kluytmans, 2017; Rau et al., 2018). However, the description of the *mcr* gene in *Acinetobacter* spp. revealed that this gene represents a real threat for rapid dissemination of resistance to the last resort therapeutic option available to face infections caused by gram-negative MDR bacteria (Hameed et al., 2019; Ma et al., 2019; Martins-Sorenson et al., 2020).

In Brazil, *Acinetobacter* spp. have exhibited elevated carbapenem resistance rates, remaining susceptible to polymyxins (Gales et al., 2012; Dias et al., 2016; Leite et al., 2016; Rossi et al., 2017). In the present study, we aimed to perform a phenotypic and molecular comparison of carbapenem-resistant *A. baumannii* strains exhibiting extensively drug-resistant (XDR) profile (Magiorakos et al., 2012) isolated from inpatients at a teaching hospital to determine whether there was an outbreak of a specific clone and to investigate the molecular mechanisms underlying the polymyxin resistance development.

## Materials and Methods

### Bacterial Isolates and Antimicrobial Susceptibility Testing

**FIGURE 1:**
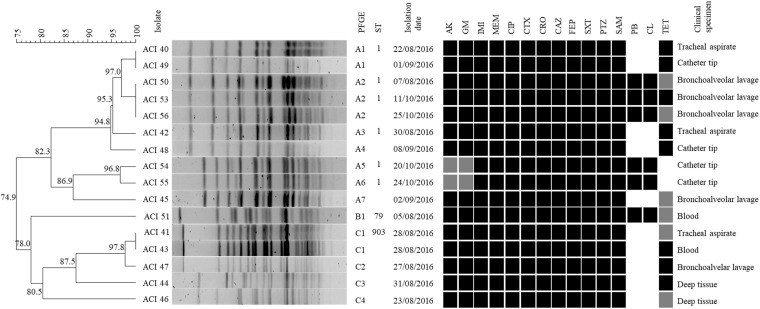
Genotypic and phenotypic characteristics of the A. baumannii isolates. From left to right, the figure shows the PFGE dendrogram with the percentage of genetic similarity, the *A. baumannii* isolates, PFGE subtype definition, sequence type (ST) according to Pasteur MLST scheme, isolation date, susceptibility profile, and clinical specimen. The following antimicrobials were tested: AK, Amikacin; CN, Gentamicin; IMI, Imipenem; MEM, Meropenem; CIP, Ciprofloxacin; CTX, Cefotaxime; CRO, Ceftriaxone; CAZ, Ceftazidime; FEP, Cefepime; SXT, Trimethoprim-sulfamethoxazole; PTZ, Piperacillin-tazobactam; SAM, Ampicillin-sulbactam; PB, Polymyxin B; CL, Colistin; TET, Tetracycline. Black, gray, and white squares represent resistant, intermediate, and susceptible, respectively.

We studied sixteen *Acinetobacter baumannii* strains recovered from 14 patients admitted to a teaching hospital in Minas Gerais state, in Southeastern Brazil (Figure 1). This was an observational and non-interventionist study using bacterial samples only, which did not require consent procedures, and was approved by the collegiate of the teaching, research and extension unit—NEPE/HRTN ethical committee (NEPE 26/2017). Serving an average of 120 patients per day in the emergency and maternity wards, this teaching hospital has 345 beds distributed in the following wards: 111 belong to the emergency, 96 to the medical clinic, 72 to surgery, 35 to intensive care unit, and 31 to maternity.

The isolates were stored in tryptic soy broth containing 40% glycerol at −80°C, and cultured on Brain Heart Infusion (BHI) agar plates at 37 °C for 24 h before performing the tests. Bacterial identification and antimicrobial susceptibility tests were determined using the Vitek 2 Compact (bioMérieux, France). Additionally, susceptibility to amikacin, gentamicin, imipenem, meropenem, ciprofloxacin, piperacillin-tazobactam, ampicillin-sulbactam, cefotaxime, ceftazidime, trimethoprim-sulfamethoxazole, and tetracycline was determined by the disk diffusion method, according to the Clinical and Laboratory Standards Institute guidelines (Clinical and Laboratory Standards Institute, 2018). Minimal inhibitory concentrations (MICs) for colistin and polymyxin B (purchased from GoldBio, United States, and Sigma-Aldrich, United States, respectively) and tigecycline (Wyeth, United States) were determined in duplicate using the broth microdilution method (Eucast, 2017; Clinical and Laboratory Standards Institute, 2018). It is important to note that the European Committee on Antimicrobial Susceptibility Testing (EUCAST) indicates that there is insufficient evidence that *Acinetobacter* species are a good target for therapy with tigecycline and that we should report the MIC value without an interpretation.

Multidrug-resistant (MDR) and extensively drug-resistant (XDR) profiles were defined using previously established criteria (Magiorakos et al., 2012). Because not all antibiotics suggested in the criteria mentioned above were available for use in this hospital, the strains were considered locally pan drug-resistant (_L_PDR) when resistant to all antibiotics tested in this study.

In addition, we determined the MIC to chlorhexidine gluconate by broth microdilution method using cation-adjusted Mueller-Hinton broth, since patient bathing with 2% chlorhexidine is a protocol to control the spread of MDR pathogens in this hospital. For this, we diluted 20% (w/v aqueous solution) chlorhexidine digluconate (Neon Comercial Reagentes Analíticos, Brazil) to obtain final concentrations ranging from 256 to 1 mg/L.

### Identification of *A. baumannii* Species by PCR

For PCR amplification, the clinical strains genomic DNA was mechanically extracted, following an adapted methodology previously described (Palazzo et al., 2007). *A. baumannii* species was confirmed by detection of the *bla*_*OXA–51–like*_ gene. Amplification of this gene was performed with primers previously described (Sohrabi et al., 2012), in a final volume of 25 μL, containing 100 ng of genomic DNA, 0.2 μM of each primer, 2.5 mM of each dNTP, 2.0 mM of MgCl_2_ and 1 U Taq DNA Polymerase (Cellco, Brazil), under the following cycling conditions: initial denaturation (95°C/1 min), 35 cycles of denaturation (95°C/30 s), annealing (57.5°C/30 s) and extension (72°C/1 min), and a final extension (72°C/2 min) in a thermal cycler (C1000, Bio-Rad Laboratories Inc., United States). The PCR products were separated by 1.5% agarose gel electrophoresis, stained with 0.01% SYBR Safe (Invitrogen, Life Technologies, United States), and visualized using the ChemiDoc XRS imaging system (Bio-Rad Laboratories Inc., United States).

### Pulsed-Field Gel Electrophoresis

The strains genetic relatedness was observed by DNA macrorestriction with 30 U of ApaI enzyme (New England BioLabs Inc., United States) followed by pulsed-field gel electrophoresis (PFGE), as previously described (Durmaz et al., 2009). The electrophoresis was run with 0.5X TBE buffer at 14°C, with 6 V/cm^2^, for 20 h and linear ramping, with initial and final switch times of 5 and 30 s, respectively, using a CHEF Mapper system (CHEF Mapper XA Pulsed Field Electrophoresis System—Bio-Rad Laboratories Inc., United States). After electrophoresis, the gel was stained with 0.01% SYBRSafe (Invitrogen, Life Technologies, United States) in 0.5x TBE buffer for 1 h, followed by visualization using the ChemiDoc XRS (Bio-Rad Laboratories Inc., United States).

We compared the DNA band profiles using the *Bionumerics* software (version 7.6, Applied-Maths, Belgium) with 0.5% optimization and 1.25% tolerance parameters, with cluster analysis done by the unweighted pair group method using the arithmetic average (UPGMA). Isolates with 100% similarity by PFGE were considered indistinguishable and clustered in the same pulsotype and subtype; isolates with similarity 80% were considered closely related and clustered in the same pulsotype, but different subtypes; isolates with similarity <80% were considered possibly related and are classified as belonging to different pulsotypes.

### Whole-Genome Sequencing, MLST, and Resistome Analysis

For whole-genome sequencing, the genomic DNA of ACI40, ACI42, ACI50, ACI51, ACI53, ACI54, and ACI55 strains was extracted according to the manufacturer instructions using the DNeasy blood and tissue kit (Qiagen GmbH, Germany). Libraries were prepared from 1 ng total DNA using a Nextera XT Sample Preparation Kit (Illumina, United States), with modifications for 2x 250 bp paired-end sequencing. Samples were sequenced using MiSeq or NextSeq platforms (Illumina, United States). CLC Genomics Workbench v.10.1.1 (QIAGEN, Denmark) was used for *de novo* genome assembly. Contigs were annotated through the NCBI Prokaryotic Genome Annotation Pipeline, and genomes were deposited at DDBJ/EMBL/GenBank. For *A. baumannii* strains ACI40, ACI42, ACI50, ACI53, ACI54, ACI55, and ACI51, sequence types (STs) were determined using the MLST 1.8 online tool from the Center for Genomic Epidemiology^1^.

For the ACI41 strain, the DNA was extracted and submitted to PCR amplification using MLST primers indicated by the Pasteur scheme^2^. PCR products were sequenced using the Sanger method (Diancourt et al., 2005). Chromatograms were checked using Vector NTi (Invitrogen, United States) and submitted to the Pasteur database in order to obtain the sequence type (ST), as determined by the combination of alleles identified^3^. Resistome of strains ACI40, ACI42, ACI50, ACI53, ACI54, ACI55, and ACI51 was predicted by using ResFinder 3.2^4^ and the Comprehensive Antibiotic Resistance Database (CARD)^5^. The presence of mutations associated with polymyxin resistance was investigated by the alignment of *eptA, pmrCAB, adeRS, lpsB*, and *lpxACD* genes (Hood et al., 2013; Lesho et al., 2013; Moffatt et al., 2019; Gerson et al., 2020; Yilmaz et al., 2020) from polymyxin-resistant (ACI50, ACI51, ACI53, ACI54, ACI55) and polymyxin-susceptible isolates (ACI40 and ACI42), whereas the genome of *A. baumannii* AB030 (NZ_CP009257.1) was used as colistin-susceptible reference strain (Loewen et al., 2014). For comparison, the *pmrB* gene of *A. baumannii* ACI56 was amplified with primers and conditions already described (Beceiro et al., 2011) in a thermal cycler (C1000, Bio-Rad Laboratories Inc., United States). The PCR product was separated by 1.5% agarose gel electrophoresis, stained with 0.01% SYBR Safe (Invitrogen, Life Technologies, United States), and visualized in the ChemiDoc XRS image system (Bio-Rad Laboratories Inc., United States). The fragment was purified by the PCR purification kit (Jena Biosciences, Germany) and sequenced by the Sanger method at the Laboratory of Biophysics Sergio Mascarenhas at the IFSC-USP. Chromatograms were checked using Vector NTi (Invitrogen, United States) and compared to the other isolates genomes’ genes. The genome sequences were submitted to GenBank, and accession numbers are as follows: WJWR00000000 (ACI40), WJWS00000000 (ACI42), PNFN00000000 (ACI50), PNJH00000000 (ACI51), WJWT00000000 (ACI53), PNJI00000000 (ACI54), and PNFO00000000 (ACI55).

### Determination of the Doubling Time

The bacteria ACI40 and ACI50 were incubated in Mueller Hinton Cation Adjusted broth at 37°C for 18 h. The suspension was adjusted to OD_600_ of 0.08–1.0, and 0.2 ml were transferred to each well in the microplate. The growth was measured by absorbance (OD_600_) using the spectrophotometer SpectraMax M5 (Molecular Devices). For growth curve and doubling time determinations, the OD vs. time was plotted for each strain in the exponential growth phase. The doubling times were then calculated as follows: [(t2 - t1) × log 2]/(log OD_600_ at t2 - log OD_600_ at t1), where t1 is sampling time 1 and t2 is sampling time 2. Two independent experiments were carried out in 6 replicates. The doubling time data were compared by ANOVA single factor.

### Biofilm Formation

The quantitative biofilm formation (biofilm mass) of *A. baumannnii* clinical strains was evaluated according to Qin et al. (2014) with modifications. Briefly, an isolated colony from a fresh culture was inoculated in 35 mL of BHI broth with 0.75% glucose and incubated (37°C for 24 h). After that, this culture was centrifuged (4,100 rpm/4°C/10 min), the pellet was resuspended in 0.5 mL of BHI broth + 0.75% glucose, and 0.05 ml of this suspension was added into 0.45 ml of phosphate-buffered saline (PBS), to reach the OD_600_ = 1.0. This adjusted suspension was then diluted (1:40) in BHI broth +0.75% glucose, and 0.2 ml was added to the wells of a flat bottom 96-wells microplate in triplicate. *Staphylococcus epidermidis* ATCC 35984 (good biofilm-forming strain) and *Staphylococcus epidermidis* ATCC 12228 (non-biofilm-forming strain) were used as positive and negative controls, respectively. Fresh BHI broth + 0.75% glucose was considered as blank. The microplate was incubated at 37°C during 24 h, the culture medium was removed, the wells were washed thrice with PBS, and let dry at room temperature. Then, 0.2 ml of crystal violet (0.2%) was added to each well to stain the biofilms for 15 min, and the wells were washed thrice with PBS. After that, 0.2 ml of ethanol:acetone solution (80:20) was added, and the microplates were shaken for 1 min. After homogenization, 0.04 ml of each well’s content was added to 0.16 ml of ethanol:acetone solution in a new microplate, and the absorbance was measured at OD_595_ in the microplate reader SpectraMax M5 (Molecular Devices, United States). The negative and positive controls (*S. epidermidis* ATCC 12228 and *S. epidermidis* ATCC 35984) were compared using the two-tailed *t*-test, for which the result was considered acceptable when the *p-*value was lower than 0.05. To determine if the isolates produced a significant amount of biofilm, they were compared by the two-tailed *t*-test with the negative control *S. epidermidis* ATCC 12228, being considered statistically different from the negative control, and therefore biofilm formers, when the *p*-value was lower than 0.05. For these cases, the percentage of biofilm formation in comparison with the negative control was calculated.

## Results

### Extensively Drug-Resistant *A. baumannii* ST1 Spread Among Patients

All isolates were confirmed as *A. baumannii* by the presence of the species-specific *bla*_*OXA–51–like*_ gene. The MICs of colistin, polymyxin B, and tigecycline are in Table 1. Six out of 16 *A. baumannii* isolates were resistant to both polymyxins representing a 37.5% incidence of polymyxin resistance in the study period. Of these polymyxin-resistant strains, only one was susceptible to tetracycline, and another was susceptible to tetracycline and tigecycline. Susceptibility to tigecycline was determined according to PK/PD breakpoints for non-species related (Eucast, 2017). Considering the parameters of Magiorakos et al. (2012), all the analyzed strains are XDR. Four of them were non-susceptible to all the 16 different antimicrobials tested, belonging to nine different antimicrobial categories, and were classified as _L_PDR due to the hospital antimicrobial availability. The chlorhexidine MIC of all isolates ranged from 16 to 32 mg/L (Table 1).

**TABLE 1:** MIC values of antibiotics and chlorhexidine for *A. baumannii* isolates.

**Isolates**	**MIC (mg/L)_a_**			
	**Colistin**	**Polymyxin B**	**Tigecycline**	**Chlorhexidine**
ACI40	0.5	0.25	2	32
ACI41	0.5	0.25	2	32
ACI42	0.25	0.25	2	32
ACI43	0.25	0.25	2	32
ACI44	0.5	0.25	1	32
ACI45	0.25	0.25	1	16
ACI46	0.5	0.25	2	32
ACI47	0.5	0.25	2	32
ACI48	0.5	0.25	2	32
ACI49	0.5	1	2	32
ACI50	**>128**	**128**	2	16
ACI51	**>128**	**64**	2	16
ACI53	**>128**	**>128**	8	32
ACI54	**>128**	**64**	1	16
ACI55	**128**	**16**	0.25	16
ACI56	**>128**	**>128**	2	16

**^*a*^Resistance in bold.**

Figure 1 shows the genetic similarity between the 16 *A. baumannii* strains by PFGE. Pulsotype A is the most prevalent among the *A. baumannii* strains isolated in this hospital (62.5%), followed by pulsotypes C and B (31.2 and 6.2%, respectively).

The strains clustered in the pulsotype A include seven different subtypes (A1–A7), 40% (*n* = 4) isolated from bronchoalveolar lavage, 40% (*n* = 4) from catheter tips, and 20% (*n* = 2) from tracheal aspirates. There are some indistinguishable strains clustered in the pulsotype A: two strains clustered in the subtype A1 (ACI40 and ACI49), and three strains clustered in the subtype A2 (ACI50, ACI53, and ACI56). The subtype A1 strains were isolated from different dates and clinical specimens (Figure 1), patients and units (data not shown), but both of them showed the same antimicrobial susceptibility profile (Figure 1). The subtype A2 strains were non-susceptible to all the antimicrobials evaluated in this study (Figure 1). Furthermore, they were all isolated from bronchoalveolar lavage (Figure 1) from different patients in the same intensive care unit (ICU) (data not shown), but at different dates (Figure 1).

The ACI51 was the first strain temporarily isolated during the study period and the only strain belonging to the pulsotype B. It was isolated from blood and showed resistance to the polymyxins (Figure 1), so it was the first polymyxin*-*resistant *A. baumannii* strain detected in this hospital. The other five polymyxin-resistant isolates (ACI50, ACI53, ACI54, ACI55, and ACI56) were clustered in the pulsotype A (Figure 1).

Pulsotype C consists of four subtypes (C1–C4), being all of them susceptible to polymyxins. They were all isolated within eight days, except for two indistinguishable strains (ACI41 and ACI43) clustered in the subtype C1 that were isolated on the same date in different clinical specimens (Figure 1) from the same patient admitted to the ICU (data not shown). These data suggest this patient had a severe disseminated infection caused by one polymyxin susceptible *A. baumannii* clone.

Of the representative isolates with their ST determined, those from pulsotype A belong to ST1 and ST405, and the pulsotype B isolate belongs to ST79 and ST233 (Pasteur and Oxford MLST schemes, respectively). One representative of pulsotype C1 belongs to the ST903 (Pasteur MLST scheme) (Figure 1).

### Molecular Basis for the Resistance Phenotype of the *A. baumannii* Strains and Fitness Comparison

The genomes coverages varied from 128x to 251x, and the details of contigs and GenBank accession numbers are in Supplementary Table S1. The acquired resistance genes detected in the draft genomes by Resfinder are in Table 2.

**TABLE 2:** Resistome and mutations related to polymyxin resistance identified in the sequenced genomes of *A. baumannii* strains.

**Characteristics**	***Acinetobacter baumannii* strains**						
	**ACI40**	**ACI42**	**ACI50**	**ACI53**	**ACI54**	**ACI55**	**ACI51**
Polymyxin susceptibility	S	S	R	R	R	R	R
MLST (ST)	ST1	ST1	ST1	ST1	ST1	ST1	ST79
**Resistome**							
Aminoglycosides	*aac(6′)Ib3, aph(3″)-Ib, aph(3′)-II, aph*(6)*-Id*	*aac(6′)Ib3, aph(3″)-I, aph(3′)-II, aph*(6)*-Id*	*aac(6′)Ib3, aph(3″)-Ib, aph*(6)*-Id*	*aac(6′)Ib3, aph(3″)-I, aph(3′)-II, aph*(6)*-Id*	*aac(6′)Ib3, aph*(6)*-Id*	*aac(6′)Ib3, aph(3″)-Ib, aph*(6)*-Id*	*aph(3″)-I, aph*(6)*-Id, aadA1*
β-lactams	*bla*_*OXA–69*_, *bla*_*OXA–23*_, *bla*_*ADC–25*_, *bla*_*TEM–116*_	*bla*_*OXA–69*_, *blaOXA-23, blaADC-25, blaTEM-116*	*blaOXA-69, blaOXA-23, blaADC-25*	*blaOXA-69, blaOXA-23, blaADC-25, blaTEM-116*	*blaOXA-69, blaOXA-23, blaADC-25*	*blaOXA-69, blaOXA-23, blaADC-25*	*blaADC-25, blaOXA-65, blaOXA-72, blaTEM-1A*
Fluoroquinolones	*aac(6′)Ib-cr*	*aac(6′)Ib-cr*	*aac(6′)Ib-cr*	*aac(6′)Ib-cr*	*aac(6′)Ib-cr*	*aac(6′)Ib-cr*	–
Macrolides			*mph(E), msr(E)*	*mph(E), msr(E)*	*mph(E), msr(E)*	*mph(E), msr(E)*	*mph(E), msr(E)*
Phenicols	*floR*	*floR*	*floR*	*floR*	*floR*	*floR*	*floR*
Sulfonamides	*sul2*	*sul2*	*sul2*	*sul2*	*sul2*	*sul2*	*sul2*
Trimethoprim	–	–	–	–	–	–	*dfrA1*
Tetracyclines	–	–	–	*tet*(39)	–	–	
*pmrA* mutations	–	–	–	–	–	–	G244A/Asp82Asn
*pmrB* mutations	–	–	C509T/P170L	C509T/P170L	C695T/T232I	C695T/T232I	C695T/T232I
*pmrC* mutations	–	–	G374A/R125H	G374A/R125H	G374A/R125H	G374A/R125H	G374A/R125H

In addition to the *aac(6′)Ib-cr* gene conferring quinolone resistance, CARD showed that all isolates have mutations leading to the Ser81Leu amino acid substitution in GyrA. The *parC* gene of the ST1 isolates also has mutations leading to Ser84Leu, Val104Ile, and Aps105Glu amino acid substitutions. ACI51 has only Val104Ile and Aps105Glu amino acid substitutions in ParC.

The tigecycline-resistance gene codifying TetX was absent in all isolates. AdeABC efflux pump and its two-component system AdeSR, both involved in multidrug resistance, including tigecycline (Ruzin et al., 2010), were present in all draft genomes. Only ACI54 and ACI55 presented the nucleotide substitution G40A in the *adeS* gene leading to Ala14Thr substitution in AdeS.

Specifically, all variants already known of the *mcr* plasmid-mediated gene and the *eptA* gene were absent in the genomes analyzed regarding polymyxin resistance.

We extracted the genes that were already reported by others as involved in polymyxins resistance *lpxACD* and *pmrCAB* from draft genomes for a detailed analysis (Moffatt et al., 2010; Lesho et al., 2013; Gerson et al., 2020). We compared the genes of the polymyxin-resistant isolates (ACI50, ACI51, ACI53, ACI54, and ACI55) plus the susceptible ACI42 (ST1) to those of the susceptible isolate ACI40 (ST1). Because some amino acid substitutions are lineage-related, we also compared the proteins codified from ACI51 (ST79) genes to those of *A. baumannii* AB030 (ST79). The mutations detected are described in Table 2.

To investigate whether all strains with higher polymyxin B MIC (ACI50, ACI53, and ACI56) had the PmrB substitution Pro170Leu (Poirel et al., 2017), the *pmrB* gene of ACI56, which did not have the draft genome sequenced, was PCR-amplified and sequenced by Sanger. As suspected, we found the same Pro170Leu substitution.

ACI40 (subtype A1) and ACI50 (subtype A2) were the only pulsotype A strains isolated from the same patient, from tracheal aspirate and bronchoalveolar lavage, respectively. They were closely related (97% similarity) by PFGE, but ACI40 was susceptible, and ACI50 was polymyxin-resistant. Comparing their genomes, we found 39 mutations leading to amino acid substitutions in 30 coding regions, including those already cited in *pmrB* and *pmrC* in ACI50 (Supplementary Table S2). We compared these coding regions to those in the other polymyxin-susceptible and resistant strains genomes sequenced in this study plus the polymyxin-susceptible ST79 AB030 strain genome. The coding regions were either absent in ST79 or were found only in ST1 polymyxin-resistant strains. Eight mutated coding regions, exclusive of ST1 polymyxin-resistant strains, codify two hypothetical proteins, an FMN-binding glutamate synthase family protein, Acyl-CoA dehydrogenase, a putative 2-aminoethyl phosphonate ABC transporter substrate-binding protein, polysaccharide biosynthesis tyrosine autokinase, fumarylacetoacetate hydrolase, and an amidohydrolase family protein (Supplementary Table S2). In addition, we compared the growth curves of the two isolates (Supplementary Figure S1) and observed that the polymyxin-resistant ACI50 strain showed longer doubling time (176 ± 17 min) compared with that of the susceptible strain ACI40 (148 ± 1), with *p* < 0.005 determined by the single-factor ANOVA.

### Biofilm Formation Is Not Enhanced Among the Polymyxin-Resistant Strains

**FIGURE 2:**
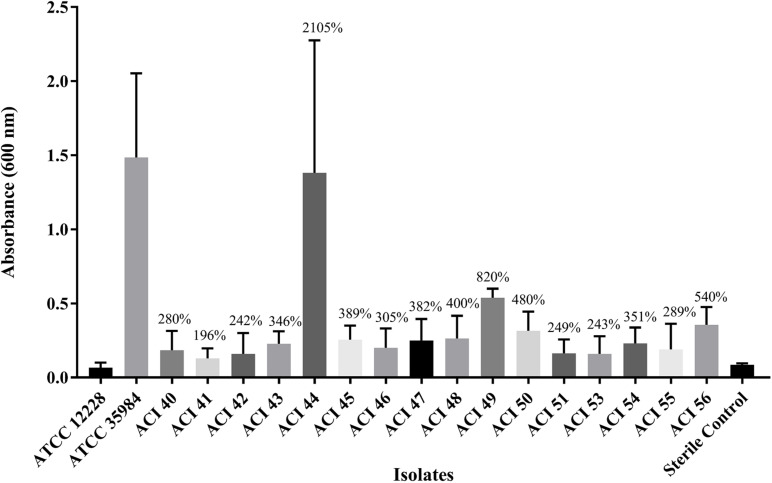
Comparison of biofilm mass formed by *A. baumannii* strains (ACI) to that of *Staphylococcus epidermidis* ATCC 12228 and ATCC 35984, the non-biofilm and the good biofilm former strains, respectively.

We wanted to investigate whether any correlation between polymyxin-resistance and biofilm formation existed. Figure 2 shows that all strains were able to form biofilms, which were statistically significant compared to the non-biofilm-former strain *S. epidermidis* ATCC 12228, or even compared to the negative control using Student’s *t*-test, *p* < 0.05. Thus, we considered all *A. baumannii* isolates as biofilm formers; The only isolate capable of forming biofilm comparable to the good biofilm-forming *S. epidermidis* ATCC 35984 strain was the polymyxin-susceptible ACI44 strain (Figure 2).

## Discussion

The isolation of gram-negative bacteria resistant to polymyxin at the Brazilian teaching hospital was unusual. Since the first polymyxin-resistant *Acinetobacter baumannii* complex isolate identification in that hospital, healthcare workers closely monitored and collected the subsequent *Acinetobacter* spp. isolates during the following three months for characterizing them and determining whether there was an outbreak. In this regard, the first polymyxin-resistant *A. baumannii* was identified in August 2016, and by the end of October 2016, the other five polymyxin-resistant *A. baumannii* isolates were identified. Our results showed that all isolates have *bla*_*OXA–51–like*_ gene, thus belonging to the *A. baumannii* species. This species prevalence was expected and agreed with other studies in Brazilian hospitals (Leite et al., 2016; Castilho et al., 2017; Vasconcellos et al., 2017).

Regarding the antimicrobial susceptibility profile obtained (Figure 1), we noticed that it is quite common that the *A. baumannii* strains isolated in this hospital are XDR. In addition, if taken the reality at the local therapeutic protocol and all the antimicrobial options available to their patients into account, several isolates were classified by the hospital as _L_PDR because 25% of them were non-susceptible to all the antimicrobials available (Figure 1). Despite the reports that the *A. baumannii* strains isolated in Brazilian hospitals are MDR, the susceptibility of those isolates to the last choice drugs, such as polymyxins, still prevails (Leite et al., 2016; Sader et al., 2016; Castilho et al., 2017; Vasconcellos et al., 2017). For instance, in a Brazilian teaching hospital, Tavares et al. (2019) found MDR and XDR *A. baumannii* clones were emerging, including CC1 strains, as the second most prevalent; on the other hand, although MDR or XDR, none of the strains was resistant to polymyxin B (Tavares et al., 2019). Nevertheless, the high incidence of polymyxin resistance found in the *A. baumannii* isolates evaluated in this study (37.5%) highlights the need for constant surveillance of antimicrobials resistance, particularly to the drugs used as last resources, in all the species isolated at the hospital. The high incidence of elevated tigecycline MIC found in the strains evaluated in this study (94%) agrees with recent studies that analyzed Brazilian clinical isolates of *A. baumannii* (Cardoso et al., 2016; Vasconcellos et al., 2017), although historically *A. baumannii* Brazilian isolates are mostly susceptible to tigecycline (Leite et al., 2016; Sader et al., 2016; Castilho et al., 2017). The use of tigecycline as a treatment for *A. baumannii* MDR strains infections is still controversial or, at least, not recommended, both in monotherapy or combined therapy (Ni et al., 2016; Eucast, 2017).

Being colonized by MDR *A. baumannii* is a risk factor for being infected by this microorganism (Fan et al., 2019). Skin decolonization with chlorhexidine bathing may help prevent catheter colonization and catheter-related bloodstream infections (Fan et al., 2019). Chlorhexidine MICs of all of our isolates are in a range already described in the literature for *A. baumannii* (Biswas et al., 2019; Nor A’shimi et al., 2019) with no difference among polymyxin-resistant and polymyxin-susceptible strains. The MICs (16–32 mg/L) were lower than the patient bathing solutions concentration (2% chlorhexidine gluconate, or 20,000 mg/L), suggesting that the bathing would benefit the spread control if those strains colonized the patients’ skin. Patient bathing with chlorhexidine and ventilators decontamination improvement (use of tubes with valves, ventilators not circulating to other patients until hospital discharge, plus being sterilized) were performed simultaneously as control measurements, and this way, the healthcare workers were able to control the dissemination of the polymyxin-resistant *A. baumannii* strains.

The biofilm is an important virulence factor that can ease the persistence of the bacterial cells in the environment and the host. In the biofilm form, they can evade immune responses and resist antibiotics more than in planktonic form (McConnell et al., 2013). The relationship between biofilm-forming ability and antibiotic resistance is of great interest to clinicians and has already been established for *Pseudomonas aeruginosa*, for instance (Abidi et al., 2013), but remains unclear for *A. baumannii* (Qi et al., 2016). We wanted to verify whether a difference in biofilm formation among polymyxin-resistant and polymyxin-susceptible isolates existed. Our results show that all strains form biofilm at similar levels, but the polymyxin-susceptible strain ACI44 was the only one that formed more biofilm (Figure 2). It is not surprising to find in the literature that non-MDR *A. baumannii* isolates can form quantitatively more biofilm and more robust than MDR or XDR isolates. These resistant isolates usually are non-biofilm formers or tend to form weaker biofilms (Rodríguez-Baño et al., 2008; Qi et al., 2016). Thus, it seems that *A. baumannii* biofilms could be a mechanism for bacteria to get a better survival in the environment, especially when their resistance to antibiotics is not high enough (Qi et al., 2016).

The PFGE results showed a clonal similarity among five out of the six *A. baumannii* strains resistant to polymyxins in this hospital. The first isolate of the studied period (ACI51, subtype B1) is only possibly related to the other isolates resistant to polymyxins by PFGE (Figure 1). One should notice that the antimicrobial susceptibility profile of these strains belonging to the subtypes A2 and B1 are practically identical, which shows that antibiogram is not enough to discriminate the similarity of the strains, requiring molecular methodologies, such as PFGE, to distinguish different genotypic profiles of strains with similar phenotypes and define the occurrence of a possible outbreak. The patient’s history from whom ACI51 was isolated shows recent hospitalization in another hospital, getting worst after five days discharged and then was admitted to the Teaching Hospital, where *A. baumannii* ACI51 was isolated from blood. Therefore, we suggest this pulsotype B ST79 strain is unrelated to the hospital endemic clone (pulsotype A).

The analysis of the draft genome sequence of five out of six polymyxin-resistant *A. baumannii*, plus two susceptible strains, shed light on the polymyxin-resistance and other mechanisms involved in the strains of this study.

ACI53 presented the higher tigecycline MIC and harbored an ORF codifying a major facilitator superfamily (MFS) antibiotic efflux pump, which was identified as *tet(D)*-like by CARD. Other mutations that may confer resistance to tigecycline, besides aminoglycosides, tetracyclines, fluoroquinolones, and chloramphenicol, are those in AdeSR, controlling the AdeABC efflux system expression level (Yilmaz et al., 2020). Substitutions in AdeRS were also already reported in colistin-resistant isolates. The draft-genomes of this study showed that the *adeS* gene of ACI54 and ACI55 presented a mutation leading to the Ala14Thr substitution; these strains were among those with the lowest tigecycline MIC.

Although there was a recent report of *mcr-4.3* gene detection in an *A. baumannii* ST79 in Brazil (Martins-Sorenson et al., 2020), in the present study, the draft genome sequences excluded the *mcr* genes in the emergence of polymyxin resistance among these isolates. Indeed, *A. baumannii* rarely harbors the *mcr* genes (Bitar et al., 2019; Hameed et al., 2019; Ma et al., 2019). The most common mechanisms of polymyxin resistance are usually related to chromosomal genes, such as those resulting in the loss of lipopolysaccharide by inactivation or insertion of sequence IS*Aba11* in the *lpxA, lpxC*, or *lpxD* genes (Moffatt et al., 2010, 2011; Henry et al., 2012) or due to the addition of phosphoethanolamine to the hepta-acylated lipid A by PmrC, which is regulated by PmrAB, thus reducing the negative charge of the bacterial cells and consecutively reducing the affinity of polymyxins to their action target (Adams et al., 2009; Beceiro et al., 2011; Gerson et al., 2020).

Amino acid substitutions in the PmrCAB are known to confer colistin resistance (Lesho et al., 2013; Durante-Mangoni et al., 2015; Moffatt et al., 2019; Gerson et al., 2020) and are likely the only reason for the polymyxin resistance in this study’s strains. We observed the substitution Asp82Asn in ACI51 PmrA, and although it is in the signal receiver domain, there is no mention in the literature relating this substitution to polymyxin resistance so far. Also, no phenotypic change was observed with Asp82Asn substitution when compared to the other strains. We subjected the mutated sequence to the SIFT algorithm^6^, which showed that the Asp82Asn substitution appears deleterious to protein activity.

Pro170Leu substitution in PmrB is known to cause an impact on the polymyxin resistance. In addition to Pro170Leu, Pro170Ser and Pro170Gln also increase *pmrC* expression in polymyxin-resistant isolates (Arroyo et al., 2011; Gerson et al., 2020). The PmrB substitution Pro170Leu was present in ACI50 and ACI53. Curiously, these strains had higher polymyxin B MICs (>128 mg/L) when compared to the ACI51, ACI54, and ACI55 (from 16 to 64 mg/L), which had a Thr232Ile substitution in PmrB, instead. ACI56 strain was indistinguishable to ACI50 and ACI53 by PFGE, belonging to subtype A2, and presented higher polymyxin B MIC (>128 mg/L) as well. For this reason, we analyzed ACI56 *pmrB* gene by PCR, revealing the same mutation found in ACI50 and ACI53. Colistin MICs of these strains were all at the same level (≥128 mg/L). Thus, it appears that Thr232Ile substitution in PmrB may lead to a lower polymyxin B MIC than isolates with Pro170Leu substitution. The polymyxin B MICs in strains with Thr232Ile substitution in PmrB in strains from another study ranged from 8 to 96 mg/L (Lesho et al., 2013). According to the SIFT algorithm, although both substitutions are deleterious, the probability of Thr232Ile substitution (probability of substitution = 0.02) is higher than the probability of Pro170Leu (probability of substitution = 0.00). Therefore, the impact of the PmrB Thr232Ile substitution in polymyxin B activity needs further investigation.

All the resistant isolates had a mutation in *pmrC*, leading to the Arg125His replacement in PmrC. According to the SIFT algorithm, the Arg125His substitution appears to be tolerant to protein activity (probability of this substitution = 0.37). Amino acid substitution in this position was already described, such as Arg125Pro, which has less impact on colistin resistance than amino acid changes in PmrA or PmrB (Gerson et al., 2020).

Because ACI40 (polymyxin-susceptible) was isolated 15 days apart after ACI50 (polymyxin-resistant), both from the same patient, the same lineage (ST1), and closely related to each other by PFGE, we concluded that different ST1 strains infected the patient during the hospital stay. Even so, we compared the genome of these strains and identified 30 mutated ORFs (Supplementary Table S2), of which eight were only present in ST1 polymyxin resistant isolates. Among them, an ORF that codifies acyl-CoA dehydrogenase gained our attention because of the involvement in the fatty acid breakdown and the membrane fluidity (Overath et al., 1969; Ma et al., 2015). Whether these mutations contribute to the mechanism of polymyxin resistance or are just related to the resistant clone expanding in the hospital, we still have to verify. In addition, the doubling-time shows that ACI40 presents a faster growth than ACI50, and some of these mutations likely affect the polymyxin-resistant isolate fitness (Pournaras et al., 2014). Further studies have to clear whether mutations leading to polymyxin-resistance simultaneously affect fitness.

Our results suggest that pulsotype A ST1 strains belong to an endemic clone that expanded in this hospital with recent mutations leading to polymyxin resistance. All the patients infected by the _L_PDR strains received polymyxin B as treatment during the hospitalization period, contributing to the resistance selection. ACI51, the first polymyxin-resistant isolate identified in this hospital, belongs to ST79, and our results, along with the patient history, showed it is unrelated to the endemic clone.

A limitation of this study is that data on polymyxin-resistant *A. baumannii* came from a single center. However, the main contribution of this study is the genomic evidence showing that carbapenem-resistant *A. baumannii* belonging to the international clone ST1 can easily expand in hospital settings and acquire chromosomal mutations in PmrB (Thr232Ile or Pro170Leu) and/or PmrC (Arg125His), resulting in the selection of polymyxin-resistant lineages displaying, at least, an _L_PDR phenotype.

In conclusion, we showed the clonal expansion of XDR *A. baumannii* ST1 strains in a Brazilian teaching hospital, including four _L_PDR strains. The polymyxin resistance in this outbreak is due to chromosomal mutations, mainly with Pro170Leu substitution in PmrB. Further investigation is needed to know whether the Thr232Ile substitution in PmrB plays a role in the lower polymyxin B MICs. The transmission of these strains among patients points out the need for constant surveillance for adopting adequate measures to avoid new nosocomial outbreaks caused by XDR carbapenem-resistant *A. baumannii* strains.

## Data Availability Statement

The datasets presented in this study can be found in online repositories. The names of the repository/repositories and accession number(s) can be found in the article/Supplementary Material.

## Ethics Statement

The studies involving human participants were reviewed and approved by the Collegiate of the Teaching, Research, and Extension Unit—NEPE/HRTN (NEPE 26/2017). Written informed consent for participation was not required for this study in accordance with the national legislation and the institutional requirements. The isolates of this study were registred at SISGEN under the number A2AAE3C.

## Author Contributions

LC conducted the susceptibility tests and wrote the manuscript. AD conducted the genome sequencing and analysis and wrote the manuscript. CB conducted the genome comparison and variant calling. GR conducted the biofilm assays and growth curves. IS conducted the analysis of chlorhexidine. JD conducted the MLST by PCR. CM responsible for organizing the data of the clinical lab from Vitek 2 and collaborate with suggestions to the manuscript. EL infectologist of the Hospital and leader of the microbiology lab, who organized the project for the sample storage and collaborated with discussions regarding the transmission in the hospital. CA responsible for isolating, identifying, and storing the bacterial collection in the clinical lab. NL conducted the genome sequencing and analysis and collaborate with suggestions to the manuscript. IC principal investigator of the group who organized the project, team, and results, and wrote part of the manuscript. All authors contributed to the article and approved the submitted version.

## Conflict of Interest

The authors declare that the research was conducted in the absence of any commercial or financial relationships that could be construed as a potential conflict of interest.

## Footnotes

1 https://cge.cbs.dtu.dk/services/MLST

2 https://pubmlst.org/bigsdb?db=pubmlst_abaumannii_pasteur_seqdef

3 https://pubmlst.org/organisms/acinetobacter-baumannii

4 https://cge.cbs.dtu.dk/services/ResFinder/

5 https://card.mcmaster.ca/home

6 https://sift.bii.a-star.edu.sg/

## Supplementary Material

Supplementary Figure 1 Growth curves of ACI40 and ACI50. The data are from two independent experiments with six replicates for each strain.

Supplementary Table 1 Genome assembly statistics and accession information.

Supplementary Table 2 Mutations that were identified in the ACI50 when compared to ACI40.
