# Supplementary figures and images for: Polymyxin Resistance Among XDR ST1 Carbapenem-Resistant Acinetobacter baumannii Clone Expanding in a Teaching Hospital
Source: Front Microbiol. 2021 Mar 26;12:622704. doi: 10.3389/fmicb.2021.622704 (PMC8063854; doi:10.3389/fmicb.2021.622704)

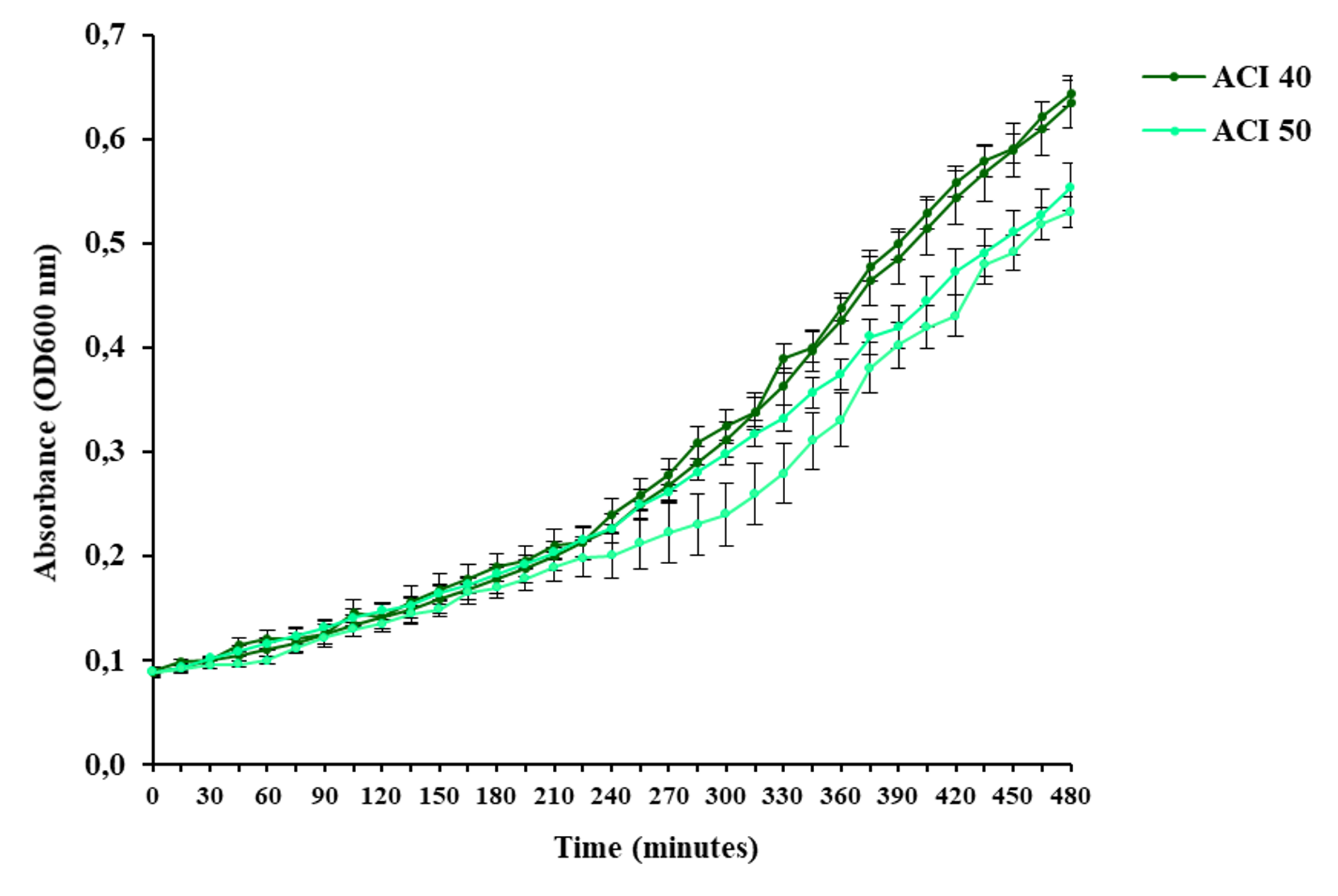

Supplement: Supplementary Figure 1 — Growth curves of ACI40 and ACI50. The data are from two independent experiments with six replicates for each strain. [file Image_1.TIFF]
