# Supplementary material for: Polymyxin Resistance Among XDR ST1 Carbapenem-Resistant Acinetobacter baumannii Clone Expanding in a Teaching Hospital
Source: Front Microbiol. 2021 Mar 26;12:622704. doi: 10.3389/fmicb.2021.622704 (PMC8063854; doi:10.3389/fmicb.2021.622704)
Supplement: Supplementary Table 1 — Genome assembly statistics and accession information. [file Table_1.DOCX]

**Table S1: Genome assembly statistics and accession information.**

| **Description** | **Coverage** | **N50** | **Number of Contigs** | **GenBank Accession** |
| --- | --- | --- | --- | --- |
| *A. baumanni* ACI40 | 251x | 97,991 | 82 | WJWR00000000 |
| *A. baumanni* ACI42 | 128x | 50,504 | 187 | WJWS00000000 |
| *A. baumanni* ACI50 | 191x | 28,811 | 252 | PNFN00000000 |
| *A. baumanni* ACI51 | 141x | 23,233 | 335 | PNJH00000000 |
| *A. baumanni* ACI53 | 230x | 73,907 | 100 | WJWT00000000 |
| *A. baumanni* ACI54 | 149x | 27,253 | 274 | PNJI00000000 |
| *A. baumanni* ACI55 | 192x | 34,783 | 234 | PNFO00000000 |

| **Table S2.** Mutations that were identified in the ACI50 when compared to ACI40. | | | | |
| --- | --- | --- | --- | --- |
| **ACI 50 Contig** | **Position (bp)** | **Mutation in ACI50** | **AA change** | **Annotation** |
| 3 | 28568 | A1396G | Lys466Glu | **Hypothetical protein** |
| 3 | 30743 | A418G | Lys140Glu | **Hypothetical protein** |
| 21 | 1059 | T353G | Ile118Ser | Tyrosine-type recombinase/integrase |
| 26 | 24223 | T316A | Ser106Thr | Hypothetical protein |
| 26 | 24167 | G372C | Glu124Asp |  |
| 26 | 24127 | A412G | Ile138Val |  |
| 26 | 24007 | A532G | Ile178Val |  |
| 26 | 23728 | G811A | Ala271Thr |  |
| 26 | 23385 | T1154C | Ile385Thr |  |
| 26 | 23383 | A1156G | Asn386Asp |  |
| 26 | 23359 | C1180T | Pro394Ser |  |
| 26 | 23180 | delGT1358_1359insAC | Asp453Gly |  |
| 26 | 23011 | T1528A | Arg510Gly |  |
| 27 | 29903 | G947A | Gly316Asp | Sensor histidine kinase KdpD |
| 29 | 2996 | C509T | Pro170Leu | *pmrB* |
| 29 | 497 | G374A | Arg125His | *pmrC* (Lipid A phosphoethanolamine transferase - PmrC) |
| 38 | 30797 | T429A | His143Gln | **FMN-binding glutamate synthase family** |
| 43 | 9174 | C500T | Pro167Leu | Diguanylate cyclase/GGDEF domain-containing protein |
| 43 | 35917 | G1019T | Gly340Val | MFS transporter |
| 48 | 12410 | G88A | Gly30Ser | Ribosome recycling factor |
| 49 | 8789 | A1124G | Tyr375Cys | **Acyl-CoA dehydrogenase** |
| 62 | 11687 | G829A | Val277Ile | HlyD family efflux transporter periplasmic adapter subunit |
| 62 | 19628 | G1067T | Gly356Val | Lipoprotein-releasing ABC transporter permease subunit LolC |
| 63 | 23771 | A972T | Glu324Asp | **Putative 2-aminoethylphosphonate ABC transporter substrate-binding protein** |
| 69 | 2930 | A1248T | Lys416Asn | **Polysaccharide biosynthesis tyrosine autokinase** |
| 89 | 17862 | G850A | Gly284Arg | Adenylate/guanylate cyclase domain-containing protein |
| 92 | 7422 | G85T | Val29Leu | Hypothetical protein |
| 94 | 21168 | A400G | Lys134Glu | **Fumarylacetoacetate hydrolase** |
| 100 | 24645 | C236A | Thr79Lys | Ribonuclease E inhibitor RraB |
| 120 | 1123 | A654T | Lys218Asn | MFS transporter |
| 124 | 6164 | A4G | Thr2Ala | High-affinity choline transporter BetT |
| 124 | 16340 | A212C | Lys71Thr | *pgaC* (poly-beta-1,6-N-acetyl-D-glucosamine synthase) |
| 130 | 24543 | 591_654del | Lys198fs | Type I secretion system protein |
| 139 | 2804 | 2542_2554del | Ala848fs | *icmF* (type VI secretion system membrane subunit TssM) |
| 161 | 3784 | 693_694insC | Lys232fs | Conjugal transfer protein TraA |
| 176 | 3558 | 550_566del | Ser184fs | *xth* (exodeoxyribonuclease III) |
| 179 | 5701 | T506C | Leu169Pro | **Amidohydrolase family protein** |
| 197 | 1270 | 56_57insACA | Gln20_Asn21insGln | Hypothetical protein |
| 228 | 444 | 765_779del | Leu256_Ile260del | *sfnG* (dymethyl sulfone monooxygenase) |

In bold, mutations found only in ST1 polymyxin resistant isolates. Fs, frame shift.
